# A Case of Life-threatening Amlodipine and Atenolol Overdose

**DOI:** 10.5005/jp-journals-10071-23181

**Published:** 2019-06

**Authors:** Sudheer Tale, Mohan Kumar, Soumitra Ghosh, Ashish Bhalla

**Affiliations:** 1-4 Department of Internal medicine, Postgraduate Institute of Medical Education and Research, Chandigarh, India

**Keywords:** Amlodipine, Atenolol, Bradycardia, Hypotension, Hyperinsulinaemia/euglycaemia therapy

## Abstract

Treating a patient of amlodipine-atenolol poisoning is nightmare for a physician. In high dose both the drugs individually cause severe bradycardia and hypotension. In combination they cause severe cardiovascular depression. Here we report a case of 66-year-old obese, hypertensive, depressed male, who presented to emergency 9 hours after consumption of 25 tablets of amlodipine-atenolol (5 mg+50 mg). On evaluation, he had refractory bradycardia, hypotension and acute kidney injury (AKI). Eventually he developed cardiac arrest. He was revived after 5 minutes of cardio-pulmonary resuscitation (CPR). He was successfully managed with gastric lavage, fluids, inotropes, atropine, isoprenaline and subsequently with calcium gluconate infusion, high-dose insulin euglycemia therapy (HIET) and lipid emulsion therapy. Glucagon infusion was also planned but it was not available. Patient hemodynamics improved and on 8th day he got the discharge. Our case exemplifies the importance of timely and aggressive management of lethal overdose of amlodipine-atenolol poisoning.

**How to cite this article:** Tale S, Kumar M, Ghosh S, Bhalla A. A Case of Life-threatening Amlodipine and Atenolol Overdose. Indian J Crit Care Med 2019;23(6):281–283.

## INTRODUCTION

Amlodipine and atenolol are widely used as fixed drug combination (FDC) for treatment of hypertension and chronic stable angina. Such tablets can be taken together with suicidal intent and treating such patients is challenging. There are two types of calcium channel blockers (CCB) dihydropyridines (amlodipine) and nondihydropyridines (verapamil), which block L-type calcium channels in vasculature and myocardium, respectively. Dihydropyridine CCB toxicity causes arterial vasodilation and reflex tachycardia, whereas non-dihydropyridine CCB toxicity causes peripheral vasodilation, decreased cardiac inotropy, and bradycardia.^1^ As the dose increases, this selectivity can be lost, and dihydropyridines may affect the myocardium and conducting system producing decreased inotropy and bradycardia.^1^ In high doses, the rate of CCB clearance decreases, prolonging the half-life and causing more toxicity.^2^ Patients ingesting more than 5–10 times the usual dose can develop signs of severe intoxication.

Atenolol is a beta blocker (BB) and these drugs competitively antagonize β-1 adrenoceptors in cardiac muscles and produce direct depressant action on the myocardium, resulting in conduction delays, bradycardia and reduced contractility with little or no effect on peripheral vasculature.^3^

Combination of amlodipine and atenolol results in additive anti-hypertensive action. The main physiological derangement being myocardial depression and hypotension, ultimately leading to tissue hypoperfusion.^4^ Overdose of FDC of these two drugs carries high mortality. Very few reports of amlodipine-atenolol overdose and its management are available in literature. We are reporting one such patient, who was successfully managed and discharged.

## CASE REPORT

**Fig. 1:**
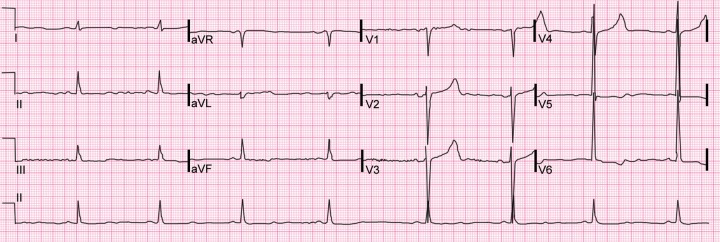
Electrocardiogram showing sinus bradycardia with heart rate of 48 bpm and tall R wave with strain pattern in precordial leads

A 66-year-old obese male, hypertensive, on treatment with amlodipine and atenolol combination (5 mg + 50 mg) and of depressive disorder, on psychiatry follow-up, was shown in local hospital at around 11.30 pm on 2/11/2016 with complaints of three episodes of vomiting followed by sudden fall while standing. Blood pressure was 100/60 mm Hg, pulse was 70 bpm. ECG was done two times but was unremarkable. He was sent home with assurance. Next morning he had similar episode and attended same hospital where he disclosed that he took 25 tablets of amlodipine-atenolol (5 mg + 50 mg) at around 8.30 pm on 2/11/2016. He was immediately referred to our hospital. While reaching the emergency triage at around 4 pm, he complained of decreased urine output with around 500 ml of urine in last 20 hours. He was drowsy with a heart rate of 48 bpm, blood pressure of 80/60 mm Hg, respiratory rate of 22 breaths per min and oxygen saturation of 88% at room air with normal systemic examination. ECG showed sinus bradycardia and tall R wave with strain pattern in precordial leads (Fig. 1). Atropine was given immediately. Gastric lavage was done. Normal saline and inotropic support with noradrenaline and dobutamine was started. Calcium gluconate (10%) 20 mL over 10 minutes was given. His laboratory investigations were normal except for blood urea and serum creatinine of 47 mg/dL and 2.0 mg/dL, respectively. Arterial blood gas analysis revealed metabolic acidosis and lactate was 2.2. Chest X-ray was normal. At around 6:30 pm, patient suddenly developed shortness of breath and palpitation followed by cardiac arrest. CPR was started immediately and was revived successfully after 5 minutes and put on ventilator. Fluid resuscitation with normal saline and inotropes were continued to maintain blood pressure. To combat bradycardia, isoprenaline tablet (10 mg three times a day), deriphylline tablet (300 mg three times a day) and nebulisation with salbutamol were started. Glucagon infusion was planned but it was not available. Repeat ECG showed heart rate of 62 bpm with corrected QT interval of 460 milliseconds and PR interval of 178 milliseconds. Ionized calcium was 0.56 mmol/L. Calcium gluconate infusion was started at a dose of 2 grams per hour. Simultaneously he was started on high-dose insulin euglycemia therapy (HIET) with infusion of high dose of regular insulin and dextrose, initially bolus doses followed by infusion along with K^+^ supplementation. Due to persistent shock and bradycardia, lipid emulsion therapy (intralipid 20%) was started, 150 mL over 1 hour followed by 25mL/h.

Patient had gradually improved. On day 2, heart rate increased to 70 bpm, BP increased to 96/60 mm Hg. Repeat ECG was normal. Urine output was 1000 mL in last 24 hours. On 3rd day, creatinine decreased to 1.2, heart rate was 80 bpm and BP was 116/70 mm Hg. Echocardiography was normal with ejection fraction of 60%. Repeat chest x-ray was normal. Creatinine was normalized by 4th day with urine output of 1.5 litres for past 24 hours. Inotropes were tapered and stopped by 5th day. He was extubated on 6th day. Psychiatry team was involved in the management since the beginning. He was discharged on 8th day with nitrazepam and quetiapine for recurrent depressive disorder in stable condition.

## DISCUSSION

The side effects of high dose of amlodipine are due to blockage of calcium channel in myocardium as well as decreased insulin levels. Amlodipine overdose causes hypotension, conduction disturbances like sinus bradycardia and varying degrees of atrioventricular block. Decreased insulin results in hyperglycemia and metabolic acidosis. Bowel infarction, stroke, hyperglycemia, and noncardiogenic pulmonary edema have also been reported.^3^ The side effect of high dose atenolol is mainly due to ion imbalance, cardiac hyperpolarization, crossing blood brain barrier to affect brain. This results in hypotension, bradycardia, low cardiac output, heart failure, cardiogenic shock and rarely, bronchospasm, hypoglycemia, acute renal failure and mesenteric ischaemia.^5^ In our case, there is combined amlodipine and atenolol poisoning, the complexity of the management is challenging. The patient developed bradycardia and hypotension. For bradycardia, initially injection atropine and later isoprenaline, deriphylline and salbutamol nebulization were given. For hypotension, isotonic normal saline and inotropes were started. As our patient had persistent bradycardia and hypotension, calcium gluconate bolus followed by infusion was started. Both BB and CCB toxicity causes intracellular hypocalcemia. Intravenous calcium supplementation is beneficial as it acts as competitive antagonist of CCB for calcium receptor and increases intracellular calcium. It modestly improves conduction, cardiac contractility, and blood pressure, thus improving both blood pressure and heart rate.^6^ As our patient had persistent hypotension, high-dose insulin euglycemia therapy with infusion of high dose of regular insulin and dextrose was considered. Potassium was also supplemented according to serum level. There is increasing evidence in support of insulin therapy while maintaining normal glucose levels in CCB poisoning.^7^ HIET has a number of mechanisms of action. Though myocardium normally utilizes free fatty acids as the primary energy source, in shock, it uses glucose as preferred energy substrate. HIET increases the intracellular transport of glucose, lactate and oxygen into myocardial cells.^8^ Also insulin has positive inotropic effect.^8^ Insulin causes vasodilatation and improves blood flow at microcirculatory level.^9^ Intravenous glucagon is the treatment of choice in beta blocker overdose. High-dose glucagon is a positive inotropic and chronotropic agent on heart muscle, which is mediated through a glucagon receptor that increases cAMP production independently of beta-adrenoceptors.^10^ It is also effective in CCB overdose.^11^ We also considered glucagon but it was not available. Due to persistent shock and bradycardia, lipid emulsion therapy (intra lipid 20%) was considered. Lipids decrease the concentration of free active drug and therefore improve myocardial activity and function.^12^ There is much evidence in favour of intravenous infusion of levosimendan in CCB and BB poisoning. It is a myocardial calcium sensitizer, which binds to cardiac troponin-C and improves the availability of calcium to actin and myosin fibers. It also has positive chronotropic action.^13^ Levosimendan infusion was considered, but our patient had improved with other measures. Gastrointestinal decontamination is advocated in CCB overdoses.^14^ In our patient, gastric lavage was done with the hope of removing drugs though much time had passed after ingestion. Other treatment options for betablocker overdose include phosphodiesterase inhibitors, cardiac pacing and hemodialysis. Cardiac pacing was not required here as he improved with medical management and had only sinus bradycardia without any conduction block. Hemodialysis is reserved for removal of renally excreted beta-blockers like atenolol, as these patients may be refractory to pharmacologic therapy.^15^ Our patient improved with medical management and urine output increased by the next day and creatinine normalized by third day. Intraaortic balloon pump, Cardiopulmonary bypass and extracorporeal membrane oxygenation are other treatment options which can be considered in refractory cases.

## CONCLUSION

Our patient took 125 mg of amlodipine along with 1250 mg of atenolol, which are very high doses. Aggressive management with multipronged approach saved our patient despite cardiac arrest and he was discharged in stable condition. In the literature, there are very few case reports of fixed drug combination of amlodipine and atenolol overdose. Our case exemplifies the importance of timely and aggressive management of lethal overdose of amlodipine-atenolol poisoning.

## References

[1] Hofer CA,, Smith JK,, Tenholder MF. (1993;). Verapamil intoxication: a literature review of overdoses and discussion of therapeutic options.. Am J Med.

[2] McAllister RG, Hamann SR,, Blouin RA. (1985;). Pharmacokinetics of calcium-entry blockers.. Am J Cardiol.

[3] DeWitt CR,, Waksman JC. (2004;). Pharmacology, pathophysiology and management of calcium channel blocker and betablocker toxicity.. Toxicol Rev.

[4] Newton CR,, Delgado JH,, Gomez HF. (2002;). Calcium and beta receptor antagonist overdose: A review and update of pharmacological principles and management.. Semin Respir Crit Care Med.

[5] Love JN,, Litovitz TL,, Howell JM,, Clancy C. (1997;). Characterization of fatal beta blocker ingestion: A review of the American Association of Poison Control Centers data from 1985 to 1995.. J Toxicol Clin Toxicol.

[6] Ghosh S,, Sircar M. (2008;). Calcium channel blocker overdose: experience with amlodipine.. Indian J Crit Care Med.

[7] Kline JA,, Leonova E,, Raymond RM. (1995;). Beneficial myocardial metabolic effects of insulin during verapamil toxicity in the anesthetized canine.. Crit Care Med.

[8] Patel NP,, Pugh ME,, Goldberg S,, Eiger G. (2007;). Hyperinsulinemiceuglycemia therapy for Verapamil poisoning: A review.. Am J Crit Care.

[9] Bechtel LK,, Haverstick DM,, Holstege CP. (2008;). Verapamil toxicity dysregulates the phosphatidylinositol 3-kinase pathway. Acad Emerg Med.

[10] Yagami T. (1995;). Differential coupling of glucagon and beta-adrenergic receptors with the small and large forms of the stimulatory G protein.. Mol Pharmacol.

[11] Doyon S,, Roberts JR. (1993;). The use of glucagon in a case of calcium channel blocker overdose.. Ann Emerg Med.

[12] Oakes JA,, Piquette C,, Barthold C. (2009;). Successful use of intravenous lipid as adjunctive therapy in a severe calcium channel antagonist poisoning.. Clin Toxicol.

[13] Varpula T,, Rapola J,, Sallisalmi M,, Kurola J. (2009;). Treatment of serious calcium channel blocker overdose with levosimendan, a calcium sensitizer.. Anesth Analg..

[14] Poggenborg RP,, Videbaek L,, Jacobsen IA. (2006;). A case of amlodipine overdose.. Basic Clin Pharmacol Toxicol.

[15] Delima LGR,, Kharasch ED,, Butler S. (1995;). Successful pharmacologic treatment of massive atenolol overdose: sequential hemodynamic and plasma atenolol concentration.. Anesthesiology.

